# Student-Led Initiatives’ Potential in the COVID-19 Response in Iran

**DOI:** 10.34172/ijhpm.2020.82

**Published:** 2020-05-29

**Authors:** Mohammad Vahidi, Laya Jalilian Khave, Ghazal Sanadgol, Dorsa Shirini, Mohammad Karamouzian

**Affiliations:** ^1^Faculty of Medicine, Shahid Beheshti University of Medical Sciences, Tehran, Iran.; ^2^School of Population and Public Health, University of British Columbia, Vancouver, BC, Canada.; ^3^Pierre Elliott Trudeau Foundation Scholar (2018) and Member of the COVID-19 Impact Committee.; ^4^HIV/STI Surveillance Research Centre, and WHO Collaborating Centre for HIV Surveillance, Institute for Futures Studies in Health, Kerman University of Medical Sciences, Kerman, Iran.

## Dear Editor,

Iran was one of the first and hardest hit countries by the coronavirus disease 2019 (COVID-19) pandemic. The first patient was first detected on February 19 and as of May 27, 2020, 14 1591 confirmed COVID-19 patients and 7564 deaths have been reported.^1^ COVID-19 strained Iran’s already resource-limited healthcare system and led to shortages in personal protective equipment and healthcare staff. Considering the need to conserve the limited available personal protective equipment for frontline staff along with the risk of medical students’ increased exposure to severe acute respiratory syndrome coronavirus 2 (SARS-CoV-2), medical students were dismissed from hospital rotations and their classes were cancelled until further notice. As senior medical interns in Shahid Beheshti University of Tehran, Iran, we were concerned not only due to loss of educational opportunities or potential occupational hazards, but also because of our non-voluntary exclusion from providing care for COVID-19 patients. As a result, we took initiative and founded the COVID-19 medical student response team (ie, *Pooyesh -e- Hamraah*) on February 22, 2020, in order to play a proactive role in Tehran’s COVID-19 response. We started as a small group of volunteer medical interns but grew rapidly and managed to recruit >100 volunteer medical interns from 12 university hospitals in Tehran. All medical interns went through a one-day personal safety and preparedness skills online crash course which was developed under the supervision of two clinical professors. Our team then developed a social media platform where educational materials were continuously shared with all volunteers and their questions and concerns were addressed and discussed. To date, over 50 senior medical interns have been trained and delivered over 2000 hours of clinical care for COVID-19 patients.

Having identified several gaps in patients’ knowledge about COVID-19 upon discharge, we designed an educational patient-friendly guideline on homecare and isolation principles; >3000 copies of which have been handed out to discharged patients in major university hospitals across Tehran. We also established a telephone-based follow-up initiative involving a team of medical students to ensure proper care and patient management post-discharge. The discharged patients were repeatedly followed up for 14 days and provided with necessary medical and psychological supports. During the follow-ups, in addition to providing care and support for the patients and their family members, we are completing a student-led research project to help document the experiences and clinical profile of discharged patients; the findings of which are summarized and shared with hospital directors to improve healthcare delivery for COVID-19 patients. To date, over 70 medical students have been trained and 816 patients have been successfully followed up through this initiative. The timeline and processes driving our initiative are illustrated in Figure.

**Figure:**
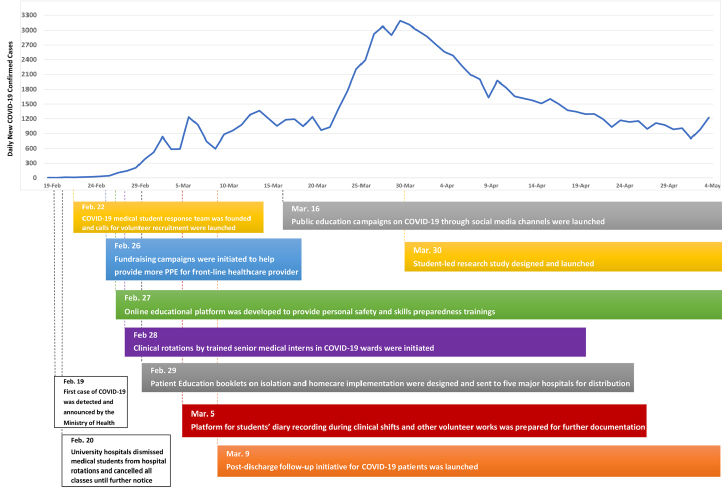
The Activity Timeline of COVID-19 Medical Student Response Team in Tehran, Iran. Abbreviation: COVID-19, coronavirus disease 2019.

As our healthcare systems adapt to the ‘new normal,’ there are opportunities for students in medical schools to assist in the COVID-19 response without having their education or safety compromised.^2-4^ Failing to acknowledge that students can significantly contribute to patient care and leaving them out of the COVID-19 response create missed opportunities for both enhanced patient care and medical education, particularly in resource-limited settings with overburdened health infrastructures. While medical schools’ hesitations over limited resources and students’ safety are understandable, medical students’ enormous potential in extending a helping hand in the midst of the worst pandemic of our times should not be overlooked. Meaningful engagement of medical students in the COVID-19 response and supporting student-led initiatives would not only help medical students become better practitioners, but also better leaders in dealing with the next phases of the COVID-19 pandemic or other future health emergencies.

## Ethical issues

Not applicable.

## Competing interests

Authors declare that they have no competing interests.

## Authors’ contributions

Conceptualization: MV, LJK, MK. Data collection: MV, LJK, GS, DS. Supervision: MK. Writing-original draft: MV, LJK, MK. Writing, review, and editing: MV, LJK, GS, DS, MK. All authors read the letter and approved the final version.

## Authors’ affiliations

^1^Faculty of Medicine, Shahid Beheshti University of Medical Sciences, Tehran, Iran. ^2^School of Population and Public Health, University of British Columbia, Vancouver, BC, Canada. ^3^Pierre Elliott Trudeau Foundation Scholar (2018) and Member of the COVID-19 Impact Committee. ^4^HIV/STI Surveillance Research Centre, and WHO Collaborating Centre for HIV Surveillance, Institute for Futures Studies in Health, Kerman University of Medical Sciences, Kerman, Iran.
